# Corrigendum: Static and Dynamic Measures of Human Brain Connectivity Predict Complementary Aspects of Human Cognitive Performance

**DOI:** 10.3389/fnhum.2018.00345

**Published:** 2018-09-07

**Authors:** Aurora I. Ramos-Nuñez, Simon Fischer-Baum, Randi C. Martin, Qiuhai Yue, Fengdan Ye, Michael W. Deem

**Affiliations:** ^1^Department of Psychology, Rice University, Houston, TX, United States; ^2^Department of Physics & Astronomy, Rice University, Houston, TX, United States; ^3^Center for Theoretical Biological Physics, Rice University, Houston, TX, United States; ^4^Department of Bioengineering, Rice University, Houston, TX, United States

**Keywords:** flexibility, modularity, resting-state fMRI, task complexity, individual differences, brain network connectivity

In the original article, there was an error. The correlation numbers stated on paragraphs three and four located under the section titled: “Relationship with Cognitive Performance” are incorrect. They should match the numbers from Table 1.

A correction has been made to the section titled: “Relationship with Cognitive Performance”, paragraphs three and four. The corrected paragraphs can be seen below:

A priori correlation analyses revealed a non-significant negative correlation between modularity measured with 400 edges and the complex composite (*r* = −0.26, *p* = 0.108). For simpler tasks, individuals with high modularity performed better, with a significant positive correlation between modularity and the simple composite (*r* = 0.34, *p* = 0.030). As might be expected, given the strong negative correlation between modularity and flexibility, there was a significant positive correlation between flexibility measured with 400 edges and the complex composite (*r* = 0.42, *p* = 0.007) and a non-significant negative correlation with the simple composite (*r* = −0.20, *p* = 0.215). The same pattern was observed at different edge densities (see Table 1).

Despite the strong correlation between flexibility and modularity, it is possible that they make independent contributions to explaining individual differences in cognitive performance. As shown in Figure 3 and Table 1, the magnitude of the correlation coefficient between modularity and the simple task composite is larger than the correlation coefficient between flexibility and simple task composite, across edge densities. The opposite pattern is true for the complex tasks. The correlation coefficient between flexibility and task performance is higher than the correlation coefficient between modularity and task performance. This pattern is partly confirmed by partial correlations analysis controlling for the effect of modularity and flexibility on task performance measured in a network with 400 edges to determine the significance of the unique contribution of each. For the simple task composite, the partial correlation for modularity was significant (*r* = 0.32, *p* = 0.046), but that for flexibility was not (*r* = 0.13, *p* = 0.44). The partial correlation for the complex task composite and flexibility was significant (*r* = 0.36, *p* = 0.022), but that for modularity was not (*r* = 0.13, *p* = 0.39).

The authors apologize for this error and state that this does not change the scientific conclusions of the article in any way.

The original article has been updated.

## Conflict of interest statement

The authors declare that the research was conducted in the absence of any commercial or financial relationships that could be construed as a potential conflict of interest.

