# Supplementary material for: Static and Dynamic Measures of Human Brain Connectivity Predict Complementary Aspects of Human Cognitive Performance
Source: Front Hum Neurosci. 2017 Aug 24;11:420. doi: 10.3389/fnhum.2017.00420 (PMC5573738; doi:10.3389/fnhum.2017.00420)
Supplement: Supplementary file 1 [file DataSheet1.DOCX]

**Supplementary Material**

**Static and dynamic measures of human brain connectivity predict complementary aspects of human cognitive performance**

Aurora I. Ramos-Nuñez^a^, Simon Fischer-Baum^a^, Randi Martin^a^, Qiuhai Yue^a^, Fengdan Ye^b,d^, and Michael W. Deem^b,c,d^

Corresponding author: rmartin@rice.edu

**1***.* **Network re-construction**

To demonstrate that the correlation between modularity and flexibility persists after using different functional and anatomical parcellations of the brain, the whole brain network was re-constructed based on methods used by others in the resting state literature (Craddock et al., 2012; Glasser et al., 2016; Gordon et al., 2016; Power et al., 2011). As shown in table 1, all the different parcellation methods yielded a negative correlation between modularity and flexibility. However, the magnitude of the coefficient was much larger using the Brodmann areas as the parcellation anatomical map.

**2.** **Relationship with task performance**

Figure 1 supplemental illustrates the relationship between modularity and task performance and flexibility and task performance during tasks varying from simple to complex processes. Simple tasks include the traffic light and the orienting effect from the Attention Network Task (ANT). Complex tasks involve the Operation Span, Digit Span, Visual Short-term Memory, the Conflict Effect from the ANT, and the Task-Shifting. For the purposes of the current research, this simple vs. complex task distinction is operationalized in the following way: complex tasks are those tasks in which executive attention and cognitive control (the ability to ignore preponderant distractors while performing correctly the task at hand) are required to properly perform the task. Simple tasks are those tasks whose performance does not depend on these operations. Because of the engagement of cognitive control, complex tasks typically require longer processing times than simple tasks. In terms of complex tasks, flexibility generally shows a larger coefficient than modularity. The opposite is true for simple tasks and modularity: modularity presents with a larger coefficient than flexibility.

**3. Sliding window parameter estimation**

We used a sliding window to compute the flexibility values. The use of a sliding window is justified by a decay of correlations in the time series beyond the width of the time window. As show in Supplemental Figure 3, correlations in the time series data decay beyond 80 s. It is for this reason that we use a sliding window of 80s in the computation of the flexibility values. The average correlation among the runs across all subjects is r=0.40 (all three p-values < 0.03).

**4. Supplementary Figure captions and Tables**

**Figure 1 Supplemental**. The relationship between modularity and flexibility with task performance represented by the magnitude of the coefficient between modularity and task performance and flexibility and task performance organized from simple (left) to complex (right). The center of the figure depicts the theoretical prediction relating performance to tasks at different levels of complexity for individuals with high and low modularity (green curve) and flexibility (red curve).

**Figure 2 Supplemental**. A comparison of flexibility across the brain between: A) an anatomical based parcellated atlas with 84 regions such as Brodmann’s Areas (BA) and B) a fuctional parcellated atlas with 100 regions from craddock et. al., 2012. The color bar on the right side of the figures represents flexibility values going from low (15 for BA atlas and 19 for Craddock atlas) to high (77 fir BA atlas and 54 for Craddock atlas).

**Figure 3 Supplemental.** We show the autocorrelation function of the fMRI time series data. The autocorrelation function of the signal in each of the 84 Brodmann areas in each of the 3 runs for each of the 52 subjects is calculated. Presented are the average of the results over Brodmann areas, runs, and subjects.

Supplementary Table 1

Modularity and Flexibility correlation coefficients from various anatomical and functional atlases

________________________________________________________________________________

r p-value

________________________________________________________________________________

BA_300_edge -0.81 p < 0.001

BA_400_edge -0.78 p < 0.001

BA_500_edge -0.74 p < 0.001

Glasser et al., 2016 -0.44 p = 0.001

Gordon et al., 2016 -0.34 p = 0.014

Power et al., 2011 -0.38 p = 0.005

Craddock et al., 2012

100 parcellations -0.49 p < 0.001

200 parcellations -0.37 p = 0.007

300 parcellations -0.42 p < 0.002

_________________________________________________________________________________

Supplementary Table 2

Modularity (M), Flexibility (F), and simple and complex task performance correlation coefficients when white matter and CSF signals were regressed out. We do not believe the white mattera and CSF signals are purely noise.

______________________________________________________________________________________________

r p-value

______________________________________________________________________________________________

M&F BA_400_edge -0.70 0.001

M BA_400_edge & simple task performance 0.23 0.161

M BA_400_edge & complex task performance -0.21 0.196

F BA_400_edge & simple task performance -0.07 0.646

F BA_400_edge & complex task performance 0.24 0.145

______________________________________________________________________________________________

Supplementary Table 3

Correlations between individual tasks and modularity and flexibility without controlling for days in between collecting behavioral measures and resting state fMRI data

_________________________________________________________________________________________________

Modularity Flexibility

_________________________________________________________________________________________________

r p-value r p-value

_________________________________________________________________________________________________

Ospan -0.410 0.011 0.335 0.040

Dspan -0.180 0.279 0.326 0.046

Conflict -0.089 0.593 0.219 0.186

VSTM -0.166 0.319 0.118 0.482

Shifting -0.026 0.875 -0.130 0.438

Orienting 0.462 0.008 -0.219 0.186

Traffic -0.190 0.254 -0.066 0.695

_________________________________________________________________________________________________

Supplementary Table 4

Correlations between individual tasks and modularity and flexibility controlling for number of days in between collecting behavioral measures and resting state fMRI data

_________________________________________________________________________________________________

Modularity Flexibility

_________________________________________________________________________________________________

r p-value r p-value

_________________________________________________________________________________________________

Ospan -0.432 0.008 0.330 0.046

Dspan -0.196 0.245 0.288 0.084

Conflict -0.090 0.595 0.224 0.182

VSTM -0.181 0.284 0.068 0.690

Shifting -0.032 0.851 -0.127 0.453

Orienting 0.431 0.008 -0.210 0.213

Traffic -0.186 0.272 -0.223 0.184

_________________________________________________________________________________________________
