# Supplementary figures and images for: Static and Dynamic Measures of Human Brain Connectivity Predict Complementary Aspects of Human Cognitive Performance
Source: Front Hum Neurosci. 2017 Aug 24;11:420. doi: 10.3389/fnhum.2017.00420 (PMC5573738; doi:10.3389/fnhum.2017.00420)

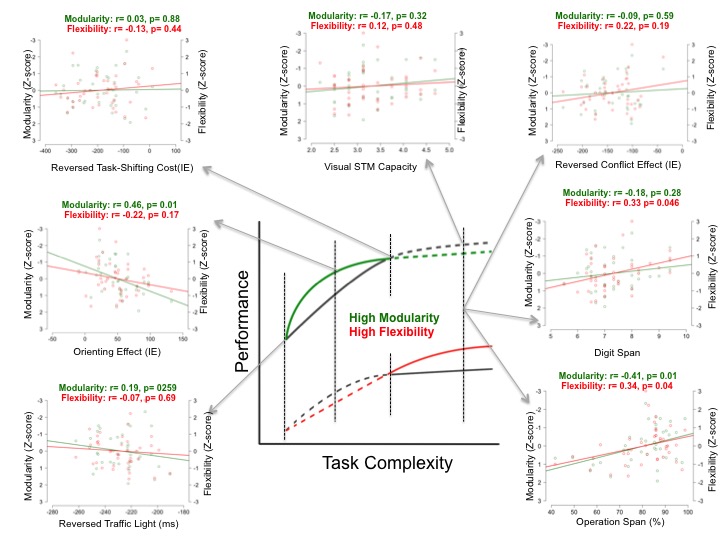

Supplement: Supplementary file 2 [file Image1.JPEG]

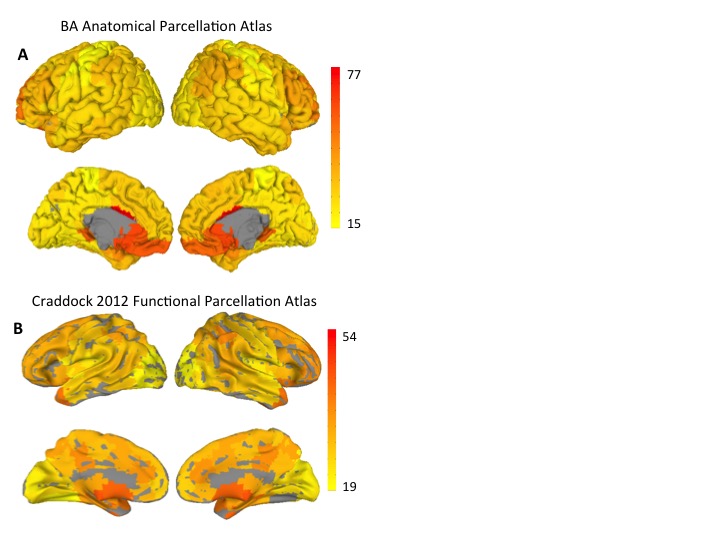

Supplement: Supplementary file 3 [file Image2.JPEG]

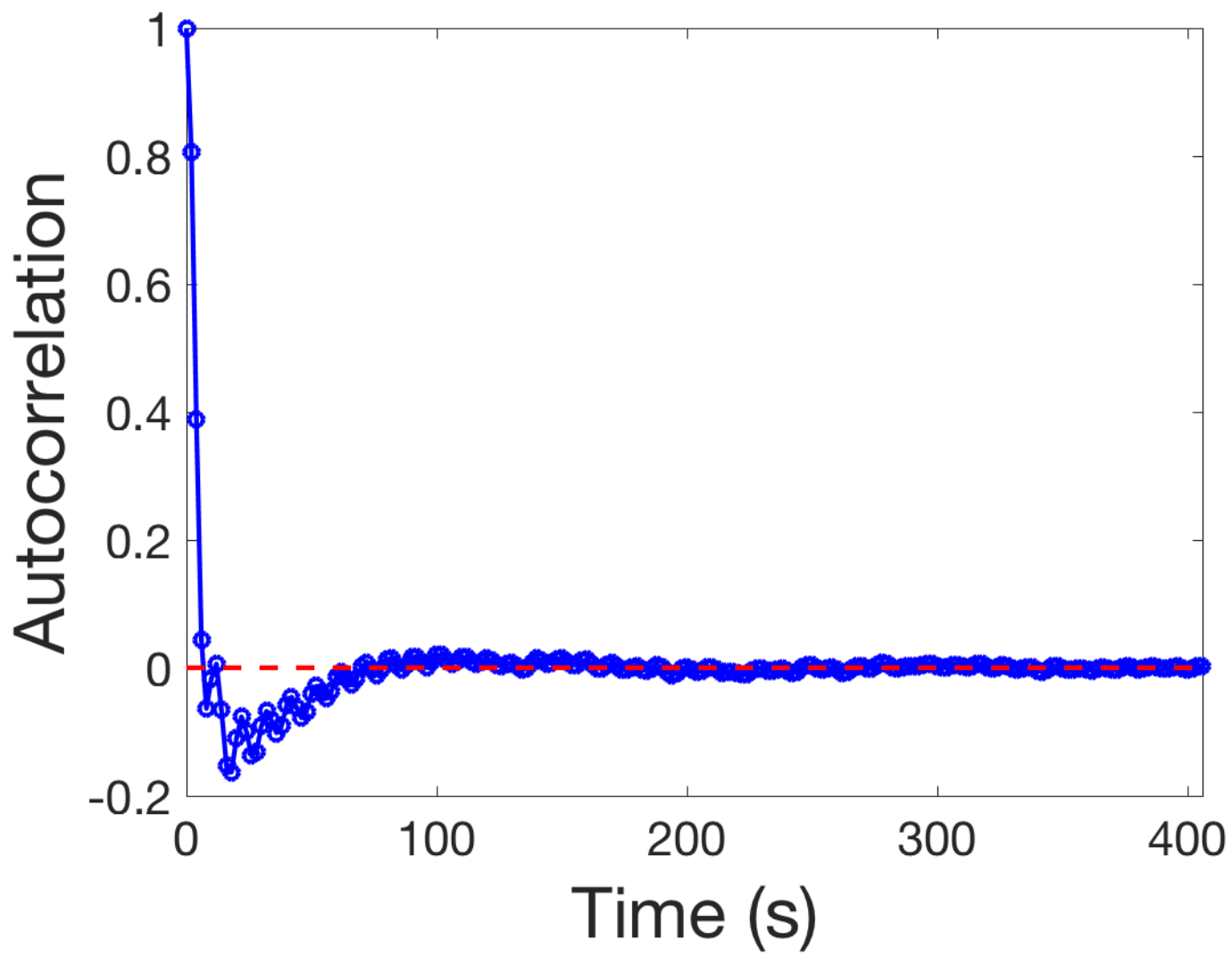

Supplement: Supplementary file 4 [file Image3.PDF]
